# Lag-3 expression and clinical outcomes in metastatic melanoma patients treated with combination anti-lag-3 + anti-PD-1-based immunotherapies

**DOI:** 10.1080/2162402X.2023.2261248

**Published:** 2023-10-04

**Authors:** Tuba N. Gide, Elizabeth C. Paver, Zarwa Yaseen, Nigel Maher, Nurudeen Adegoke, Alexander M. Menzies, Ines Pires da Silva, James S. Wilmott, Georgina V. Long, Richard A. Scolyer

**Affiliations:** a Melanoma Institute Australia, The University of Sydney, Sydney, Australia; b Charles Perkins Centre, The University of Sydney, Sydney, Australia; c Faculty of Medicine and Health, The University of Sydney, Sydney, Australia; d NSW Health Pathology, Sydney, Australia; e Tissue Pathology and Diagnostic Oncology, Royal Prince Alfred Hospital, Sydney, Australia; f Department of Medical Oncology, Royal North Shore Hospital, Sydney, Australia; g Department of Medical Oncology, Mater Hospital, Sydney, Australia; h Department of Medical Oncology, Blacktown and Westmead Hospitals, Sydney, Australia

**Keywords:** LAG-3, biomarker, immune checkpoint inhibitors, immunotherapy, melanoma

## Abstract

Lymphocyte-activation gene-3 (LAG-3), an immune checkpoint receptor, negatively regulates T-cell function and facilitates immune escape of tumors. Dual inhibition of LAG-3 and programmed cell death receptor-1 (PD-1) significantly improved progression-free survival (PFS) in metastatic melanoma patients compared to anti-PD-1 therapy alone. Investigating the utility of LAG-3 expression as a biomarker of response to anti-LAG-3 + anti-PD-1 immunotherapy is of great clinical relevance. This study sought to evaluate the association between baseline LAG-3 expression and clinical outcomes following anti-LAG-3 and anti-PD-1-based immunotherapy in metastatic melanoma. LAG-3 immunohistochemistry (clone D2G4O) was performed on pre-treatment formalin-fixed, paraffin-embedded metastatic melanoma specimens from 53 patients treated with combination anti-LAG-3 + anti-PD-1-based therapies. Eleven patients had received prior anti-PD-1-based treatment. Patients were categorized as responders (complete/partial response; *n* = 36) or non-responders (stable/progressive disease; *n* = 17) based on the Response Evaluation Criteria in Solid Tumours (RECIST). Tumor-infiltrating lymphocytes (TILs) were scored on hematoxylin and eosin-stained sections. LAG-3 expression was observed in 81% of patients, with staining in TILs and dendritic cells. Responders displayed significantly higher proportions of LAG-3+ cells compared to non-responders (*P* = .0210). LAG-3 expression positively correlated with TIL score (*P* < .01). There were no significant differences in LAG-3 expression between different sites of metastases (*P* > .05). Patients with ≥ 1% LAG-3+ cells in their tumors had significantly longer PFS compared to patients with < 1% LAG-3 expression (*P* = .0037). No significant difference was observed in overall survival between the two groups (*P* = .1417). Therefore, the assessment of LAG-3 expression via IHC warrants further evaluation to determine its role as a predictive marker of response and survival in metastatic melanoma.

## Introduction

The lymphocyte activation gene 3 (LAG-3) is an immune checkpoint receptor expressed on activated cytotoxic and helper T-cells, regulatory T-cells, natural killer cells, B cells and dendritic cells. LAG-3 interacts with its ligand major histocompatibility complex II (MHC II) on tumor cells, as well as other emerging and less characterized ligands including fibrinogen-like protein 1 (FGL-1), α-synuclein fibrils (α-syn), galectin-3 (Gal-3) and lymph node sinusoidal endothelial cell C-type lectin (LSECtin), to negatively regulate T-cell function and the immune response.^1^

Clinical trials testing LAG-3 inhibitors in combination with programmed cell death 1 (PD-1) inhibitors have shown significant improvements in progression-free survival (PFS) compared to PD-1 inhibition alone in patients with melanoma. Dual blockade of the LAG-3 and PD-1 checkpoints with the inhibitors relatlimab and nivolumab, respectively, demonstrated efficacy in a Phase I/II study including patients with metastatic melanoma who had received prior anti-PD-1/PD-L1 immunotherapies, with an objective response rate (ORR) of 12.0% in patients with only 1 prior line of anti-PD-1 therapy and 9.2% in patients with ≥ 1 line of anti – PD-(L)1-containing regimens.^2^ The randomized Phase II/III RELATIVITY-047 trial evaluated combination nivolumab + relatlimab versus nivolumab alone in previously untreated patients with metastatic melanoma, and demonstrated an improved PFS with the combination nivolumab + relatlimab compared to nivolumab alone with a 1-year PFS of 47.7% versus 36%, respectively (hazard ratio [HR] for progression or death, 0.75, *P* = .006).^3^ The overall survival was numerically improved with HR 0.80 (95% CI, 0.64 to 1.01; *P* = .059).^4^ These findings resulted in the Food and Drug Administration (FDA) approval of nivolumab and relatlimab for patients with unresectable or metastatic melanoma.^5^

Several studies have investigated the potential of LAG-3 expression as a biomarker of response or resistance to the standard-of-care anti-PD-1 therapies, including in the RELATIVITY-047 study. Early retrospective studies have conflicting findings across different cancer types. High levels of pre-treatment serum soluble LAG-3 in patients with advanced melanoma, as well as LAG-3+ T-cell infiltration in their melanoma metastases have been shown to be associated with resistance to anti-PD-1 immunotherapy.^6^ Furthermore, a high percentage of peripheral LAG-3^+^CD8^+^ T-cells was associated with poor response and significantly shorter PFS and overall survival (OS) in melanoma and urothelial cancer patients treated with anti-PD-1 monotherapy.^7^ In contrast, higher levels of LAG-3-positive CD4+ and CD8+ T-cells in peripheral blood correlated with longer PFS in patients with advanced gastric cancer treated with nivolumab.^8^ However, the association between LAG-3 expression and response to anti-LAG-3-based immunotherapy remains under investigation. The RELATIVITY-047 trial showed that the median PFS estimates were longer for patients with ≥ 1% LAG-3 expression compared to patients with < 1% expression for both treatment groups, indicating that LAG-3 expression could not be used to select the patients who would benefit from the addition of an anti-LAG-3 inhibitor to anti-PD-1 with high sensitivity or specificity.^3^

In this study, we assessed the expression of LAG-3 in the tumor microenvironment (TME) of melanoma metastases and evaluated the association between baseline immunohistochemical LAG-3 expression and clinical outcomes in patients with metastatic melanoma treated with either dual anti-LAG-3 + anti-PD-1 combination immunotherapy or triple anti-LAG-3 + anti-PD-1 + anti-CTLA-4 immunotherapy. Furthermore, we investigated the association between LAG-3 expression and clinical outcomes in a subset of patients who progressed following prior anti-PD-1-based therapy.

## Materials and methods

### Patients and specimens

This study included a cohort of 53 patients treated with combined anti-PD-1 + anti-LAG-3 ± anti-CTLA-4 immunotherapy with available baseline formalin-fixed, paraffin-embedded (FFPE) melanoma tissue (NCT03459222, NCT03470922, and NCT01968109). Patients were either anti-PD-1 treatment naïve or had progressive disease with PD-1 therapy. This study was approved by the New South Wales Department of Health Human Research Ethics Committee (Protocol no. X15–0454) and conducted in accordance with the Declaration of Helsinki. Samples were acquired with consent from the Melanoma Biospecimen Tissue Bank (HREC/11/RPAH/444). Patient response was determined using the RECIST 1.1 criteria.^9^ Responders were categorized as patients with a RECIST response of complete response or partial response, while non-responders were categorized as those with progressive disease or stable disease.

### LAG-3 immunohistochemistry

Four µm sections from FFPE metastatic melanoma specimens were heated in an oven at 65°C for 20 minutes, deparaffinized in xylene and rehydrated in graded ethanols. Antigen retrieval was performed in high pH HIER buffer (pH 9) in the Decloaking Chamber (Biocare Medical) at 110°C for 10 minutes. Staining was performed using an Autostainer Plus (Agilent). Slides were incubated with the primary rabbit monoclonal LAG-3 antibody (Cell Signalling, clone D2G4O) at a 1:50 dilution for 30 minutes. The antibody was detected using the MACH 4 Universal HRP-Polymer for 20 mins (Biocare Medical, M4U534) before visualization using the Betazoid DAB Chromogen kit (Biocare, BDB2004L) for 5 mins. Slides were then counterstained with hematoxylin and coverslipped.

### Pathological assessment and scoring

Specimens were assessed for melanoma using hematoxylin and eosin (H&E) sections to ensure ≥ 5% tumor content in each sample. Samples with less than 5% tumor content were excluded from further analysis. Tumor-infiltrating lymphocytes (TILs) were scored on H&E sections using a four-tier TIL grading scheme, as previously described.^10^ The presence and distribution of melanophages were also noted. LAG-3 expression via immunohistochemistry (IHC) was assessed by a pathologist blinded to clinical outcome (E.C.P. or N.M.). LAG-3 was evaluated on lymphocytes expressing punctate, cytoplasmic, or membranous LAG-3 (Supplementary Figure S1), as described previously.^11^ Expression on dendritic cells was not included in the overall evaluation of LAG-3. LAG-3-positive immune cells were differentiated from melanophages based on their small, dense, round nuclei and minimal cytoplasmic volume. In cases that were deemed difficult to interpret, the corresponding H&E section was referred to and the distribution of TILs and melanophages were compared. LAG-3 distribution varied between cases, with some specimens displaying LAG-3 expression only in focal ‘hotspot’ regions. Samples were classified as LAG-3-positive if the number of LAG-3+ lymphocytes was ≥ 1% of all cells.

### Statistical analysis

All statistical analyses were performed using GraphPad Prism 9.0. Fisher’s exact test and the Chi-square test were used to compare clinicopathologic parameters (Table 1). Kaplan–Meier log-rank analyses were performed to determine associations between LAG-3 expression and PFS and OS. The correlation between LAG-3 expression and TILs was assessed using Spearman’s rank correlation test. Univariable and multivariable logistic regression was utilized to analyze the relationship between baseline LDH, M stage at entry, LAG-3 and TIL score with response class. Specifically, univariable logistic regression was employed to investigate the individual associations of each predictor with response. Multivariable logistic regression was then used to determine their joint effects on the response class. Other statistical analyses involved the Mann–Whitney U test and one-way ANOVA with Dunn’s multiple comparisons test. A *P* value of less than 0.05 was considered statistically significant.

**Table 1.** Clinicopathologic characteristics of patients treated with anti-LAG-3 immunotherapy.

Patient Characteristics	Responders (*n* = 37)	Non-responders (*n* = 16)	Total (*n* = 53)	*P* value
Age (median, years)	62	67	63	–
Sex, n (%)				
Male	24 (65)	11 (69)	35 (66)	*P* > .99
Female	13 (35)	5 (31)	18 (34)	
Elevated LDH, n (%)	8 (22)	10 (63)	18 (34)	*P = .0098
BRAF V600 mutation, n (%)	12 (33)	6 (38)	18 (34)	*P* = .76
Treatment, n (%)				
Anti-PD-1 + Anti-LAG-3	28 (76)	14 (88)	42 (79)	*P* = .47
Anti-PD-1 + Anti-CTLA-4 + Anti-LAG-3	9 (24)	2 (13)	11 (21)	
Prior BRAFi, n (%)	1 (3)	5 (31)	6 (11)	*P = .0074
Prior immunotherapy, n (%)	3 (8)	8 (50)	11 (21)	*P = .0014
M stage (AJCC 8th edition), n (%)				
M0	6 (17)	1 (6)	7 (13)	UND
M1a	2 (6)	0 (0)	2 (4)	
M1b	13 (36)	2 (11)	15 (28)	
M1c	14 (39)	14 (78)	28 (52)	
M1d	1 (3)	1 (6)	2 (4)	
Response^1^, n (%)				
CR	6 (16)	0 (0)	6 (11)	UND
PR	31 (84)	0 (0)	31 (58)	
SD	0 (0)	4 (25)	4 (8)	
PD^2^	0 (0)	12 (75)	12 (23)	

Abbreviations: Anti-PD-1 – anti-programmed cell death-1; anti-LAG-3 – anti-lymphocyte activation gene-3; anti-CTLA-4 – anti-cytotoxic T-lymphocyte activation-4; LDH – lactate dehydrogenase; AJCC – American Joint Committee on Cancer; CR – complete response; PR – partial response; SD – stable disease; PD – progressive disease; % - percentage; UND – undetermined due to small numbers.

Fisher’s exact test *P* values are reported where appropriate. *P < .05.

^1^Patients were stratified into response groups based on RECIST 1.1 criteria. Patients with CR and PR were classified as responders, while patients with SD and PD were classified as non-responders.

^2^One patient with no imaging was categorized as having progressive disease based on their time to progression (<6 months) and cause of death (melanoma).

## Results

### Patient characteristics and specimens

Fifty-three patients with unresectable Stage III or Stage IV melanoma treated with anti-LAG-3 in combination with anti-PD-1 ± anti-CTLA-4 were included in this study. Forty-two patients received anti-PD-1 plus anti-LAG-3 immunotherapy, and eleven patients received anti-PD-1 + anti-CTLA-4 + anti-LAG-3 (Table 1). Patients were categorized as responders (*n* = 37), or non-responders (*n* = 16) based on RECIST 1.1 criteria as described above. At baseline, the median age was 63 years (range 43–82), 66% (*n* = 35/53) were male, and 34% (*n* = 18/53) had an elevated lactate dehydrogenase (LDH). Thirty-six percent of patients (*n* = 18/53) had a BRAF V600 mutation, 63% of which were BRAF V600E, and 21% (*n* = 11/53) had a NRAS mutation. Twenty-one percent of patients (*n* = 11/53) had received prior immunotherapies including anti-PD-1/PD-L1 monotherapy or in combination with ipilimumab. A significantly higher proportion of non-responders had elevated LDH (*P* = .0098), prior BRAF inhibition (*P* = .0074) and prior immunotherapy (*P* = .0014) compared to responders (Table 1).

### LAG-3 expression in melanoma specimens

**Figure 1.**
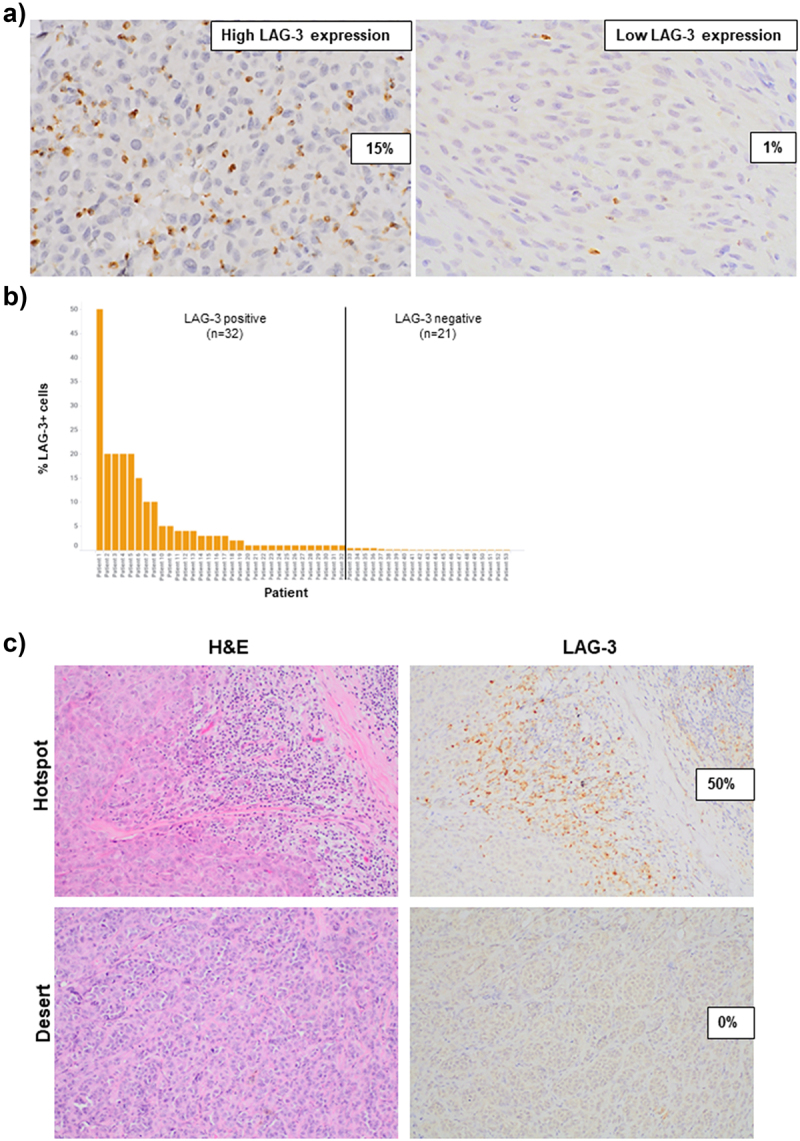
LAG-3 expression in pre-treatment melanoma specimens.

LAG-3 staining of lymphocytes was observed in 43 baseline tumor specimens (81%, *n* = 43/53) of patients treated with combination anti-LAG-3 + anti-PD-1 ± anti-CTLA-4 immunotherapy. LAG-3 staining was also observed in cells with dendritic morphology. No LAG-3 expression was seen on melanoma cells. The median percentage of LAG-3+ cells among all LAG-3-positive specimens was 3% (ranging from 1 to 50%; Figure 1a). Eighty-one percent of patients (43/53) showed < 5% LAG-3 expression. Specimens with LAG-3 staining ≥ 1% of all cells were considered LAG-3-positive, with 60% (*n* = 32/53) of patients categorized as having a LAG-3-positive immune microenvironment (Figure 1b). In a subset of cases, high LAG-3 staining was observed only in focal ‘hotspot’ areas, with the remainder of the tumor showing apparent exclusion of LAG-3+ cells (Figure 1c).

a) Representative images demonstrating high and low LAG-3 staining in a pre-treatment metastatic melanoma specimen. b) Bar graph illustrating the proportion of patients with LAG-3-positive tumors (LAG-3 ≥ 1%). c) Representative images showing hotspot regions with high LAG-3 staining and desert regions with no LAG-3 staining, which correlated with the amount of immune cells present. All images were taken at 40× magnification.

**Figure 2.**
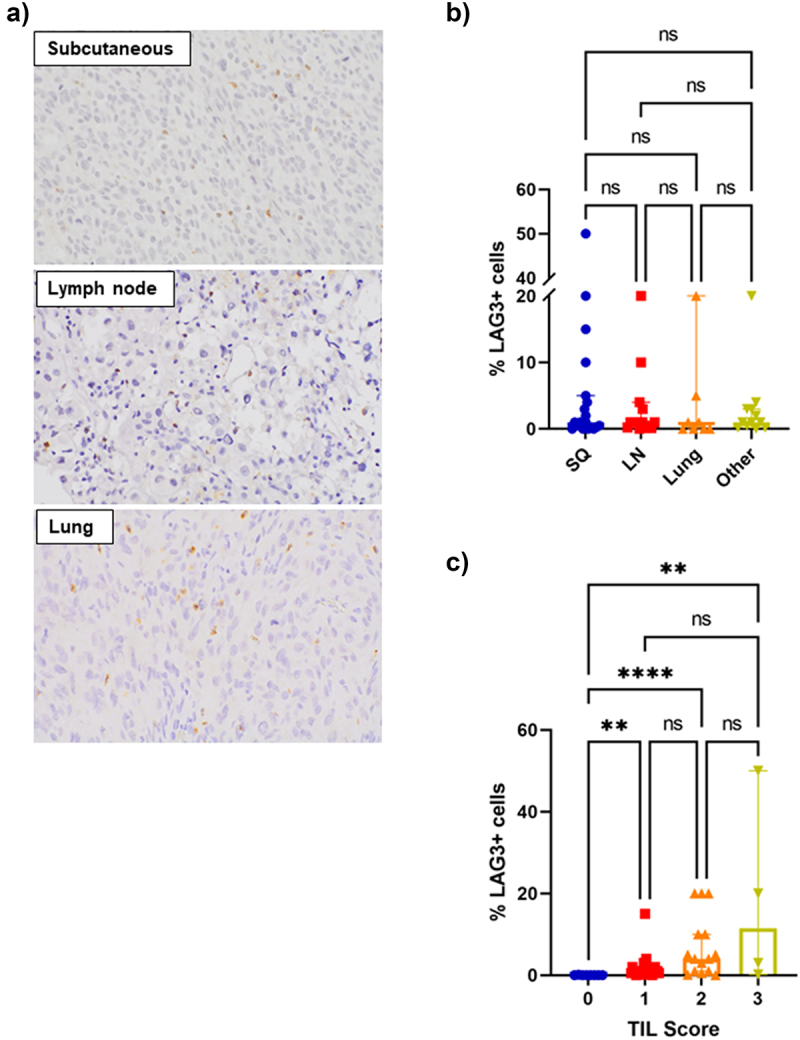
Association between LAG-3 expression, sites of disease and TILs.

Median LAG-3 expression was similar across the different sites of metastases, including subcutaneous (median = 1%), lymph node (median = 1%) and lung metastases (median = 0.5%) (Figure 2a). There were no significant differences in LAG-3 expression between the different sites of metastases (*P* = .7471) (Figure 2b).

a) Representative images (40× magnification) showing LAG-3 staining in subcutaneous, lymph node and lung metastases. b) Bar graph revealing no significant differences in LAG-3 expression between sites of metastases. c) Bar graph showing significant associations between LAG-3 expression and TIL grade. Error bars represent median ±95% CI. **P < .01, ****P < .0001, ns – non-significant, as determined by a Kruskal–Wallis test.

Tumors with a TIL grade of 3 had the highest expression of LAG-3 (median = 11.5%), followed by TIL grade 2 tumors (median = 4%) and TIL grade 1 tumors (median = 1%) (Figure 2c). LAG-3 expression in tumors of TIL grades 1–3 was significantly higher than tumors with a TIL grade of 0 (*P* < .01).

### Association of LAG-3 expression with response and survival

**Figure 3.**
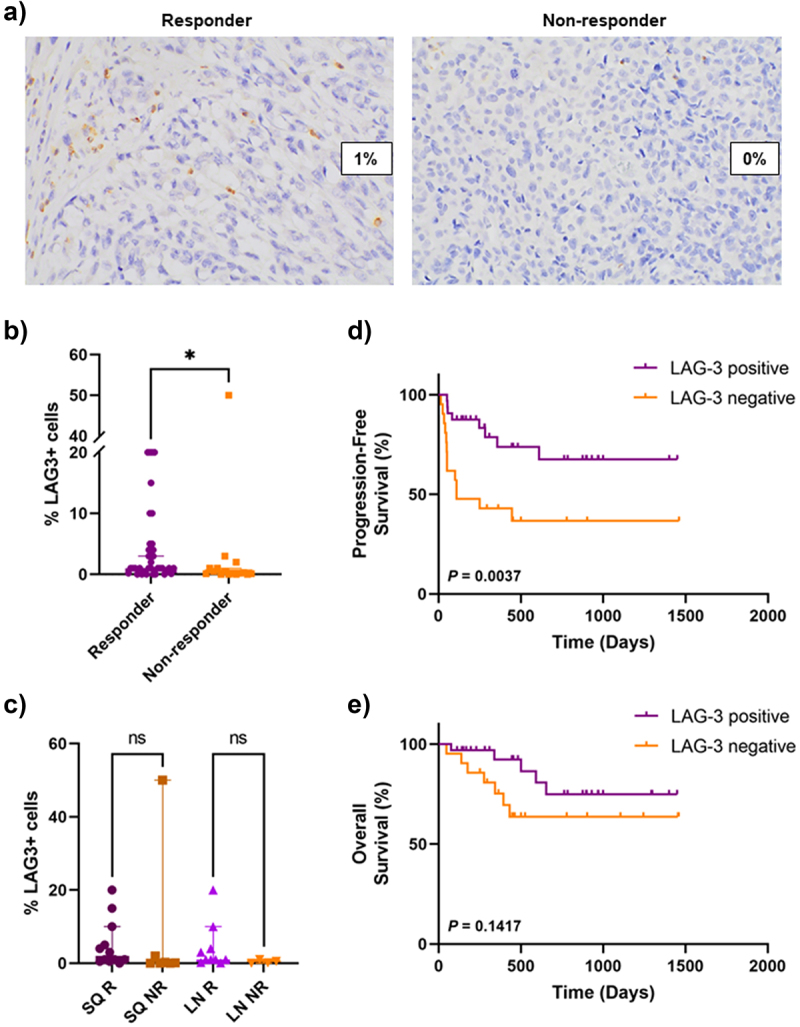
Association between LAG-3 expression and clinical outcomes.

LAG-3 expression was significantly higher in responders to anti-PD-1 + anti-LAG-3 combination immunotherapy compared to non-responders (*P* = .0210; median = 1.0% in responders vs 0.25% in non-responders) (Figure 3a,b). The response rate was 84% (*n* = 27/32) in patients with LAG-3-positive tumors compared to 48% (*n* = 10/21) in patients with LAG-3-negative tumors. There were no significant differences in LAG-3 expression between responders and non-responders within subcutaneous specimens (*P* = .0853) or within lymph node specimens (*P* = .0519) (Figure 3c).

a) Representative images (40× magnification) illustrating LAG-3 expression in responders and non-responders. b) Bar graph showing significantly higher percentage of LAG-3+ cells in responders compared to non-responders. c) Bar graph demonstrating no significant differences in the percentage of LAG-3+ cells between responders and non-responders based on site of disease. Kaplan–meier curves comparing d) progression-free survival and e) overall survival between patients with LAG-3-positive and LAG-3-negative tumors using the log-rank (Mantel-Cox) test. Error bars represent median ±95% CI. *P < .05, ns – non-significant, as determined by a Mann–Whitney U test. SQ – subcutaneous, LN – lymph node, R – responder, NR – non-responder.

To further evaluate the association between LAG-3 expression and response to anti-LAG-3 combination immunotherapy, univariate and multivariate analyses including LAG-3 status, TILs, M stage and baseline LDH were performed. In univariate analyses, LAG-3 positivity, normal baseline LDH and earlier M stage disease were associated with response (*P* = .0065, *P* = .0059 and *P* = .0048, respectively; Table 2). Following multivariate analyses, LAG-3 positivity (*P* = .0064, adj. OR = 13.594, 95%CI = 2.083, 88.726) and M stage at entry (*P* = .0137, adj. OR = .082, 95% CI = 0.011, 0.599) were associated with better response. The level of TILs present was not associated with response in either univariate or multivariate analyses (*P* = .2836 and *P* = .3894, respectively; Table 2).

**Table 2.** Univariate and multivariate logistic regression analyses for response to combination anti-LAG-3 + anti-PD-1-based immunotherapy.

	Univariate Analysis				Multivariate Analysis			
	OR	Lower CI	Upper CI	*P* value	Adjusted OR	Lower CI	Upper CI	*P* value
Baseline LDH								
Elevated	1				1			
Normal	6.042	1.681	21.718	.0059	5.581	0.984	31.665	.0522
LAG-3 status								
Negative	1				1			
Positive	5.94	1.648	21.41	.0065	13.594	2.083	88.726	.0064
M stage at entry								
M0/M1a/b	1				1			
M1c/d	0.097	0.019	0.492	.0048	0.082	0.011	0.599	.0137
TIL score								
High	1				1			
Low	0.489	0.132	1.809	.2836	2.29	0.347	15.101	.3894

Abbreviations: LDH – lactate dehydrogenase; LAG-3 – lymphocyte activation gene-3; TIL – tumor-infiltrating lymphocyte; OR – odds ratio; CI – confidence interval.

Significant *P* values are in bold.

Next, we evaluated the association between LAG-3 expression, PFS and OS. Patients with LAG-3-positive tumors had a significantly longer PFS compared to patients with LAG-3-negative tumors (*P* = .0037) (Figure 3d). The median PFS was 3.6 months for patients with LAG-3-negative tumors, while the median for patients with LAG-3-positive tumors was not reached. No significant differences were observed in OS between the two groups (*P* = .1417) (Figure 3e).

### LAG-3 expression in PD-1 refractory patients

We next examined the expression of LAG-3 in the 11 patients who had previously been treated with anti-PD-1-based immunotherapies. All specimens were taken following previous anti-PD-1-based therapy, and prior to anti-LAG-3 immunotherapy. Three patients (27%) responded to the combination anti-LAG-3 + anti-PD-1 immunotherapy, and eight patients (73%) were categorized as non-responders.

**Figure 4.**
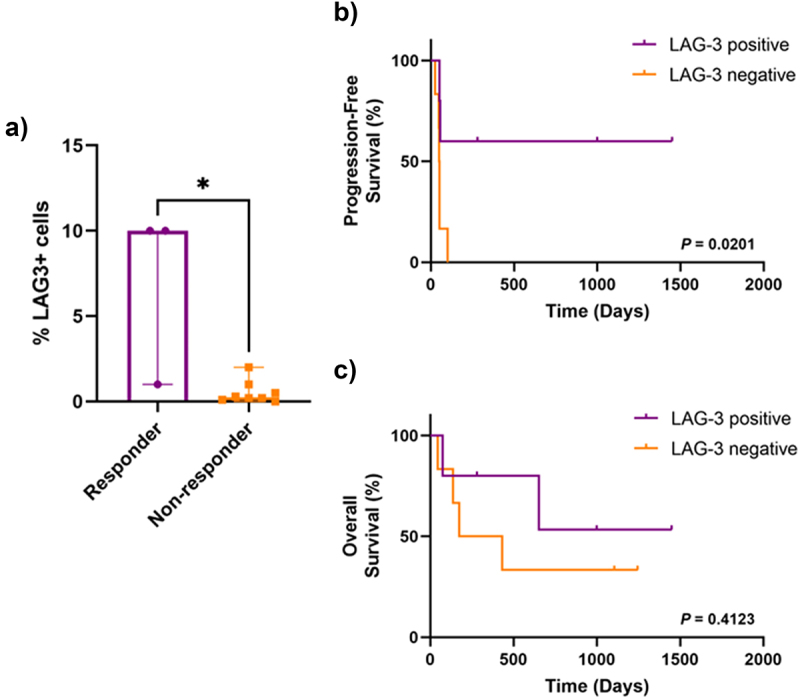
LAG-3 expression in anti-PD-1 refractory patients.

Five of 11 patients (45%) had LAG-3-positive tumors. LAG-3 expression was significantly associated with response to anti-PD-1 and anti-LAG-3 immunotherapy in PD-1 refractory patients (*P* = .0303) (Figure 4a). Similar to the larger cohort, anti-PD-1 refractory patients with LAG-3-positive tumors had a significantly longer PFS compared to patients with LAG-3-negative tumors (*P* = .0201) (Figure 4b). There was no significant difference in OS between the two groups (*P* = .4123) (Figure 4c).

a) Bar graph showing significantly higher percentage of LAG-3+ cells in responders compared to non-responders. Kaplan–Meier curves comparing b) progression-free survival and c) overall survival between patients with LAG-3-positive and LAG-3-negative tumors using the log-rank (Mantel-Cox) test. Error bars represent median ±95% CI. *P < .05, as determined by a Mann–Whitney U test.

## Discussion

In this study, we investigated baseline LAG-3 expression in metastatic melanoma specimens from patients treated with combination anti-LAG-3 + anti-PD-1 immunotherapy. Our study revealed a significant association between LAG-3 expression and response to anti-LAG-3-based immunotherapies, regardless of whether the anti-LAG-3 therapy was given first-line or after progression on anti-PD-1-based therapies. Furthermore, LAG-3 positivity strongly correlated with progression-free survival, but not overall survival. These findings may have implications for the use of LAG-3 expression as a potential biomarker of response to anti-LAG-3 immunotherapy for patients with metastatic melanoma, particularly after progression on anti-PD-1 therapy.

Given the efficacy of immunotherapies in treating different cancers including melanoma, a critical question that remains under investigation is whether the expression of specific immune checkpoints is associated with response to their blockade. CTLA-4 expression on tumor cells has been shown to be associated with response to the anti-CTLA-4 inhibitor, ipilimumab, in melanoma.^12^ Several studies have also highlighted the significant correlation between PD-L1 expression and clinical outcomes on anti-PD-1-based immunotherapies.^13–15^ However, the clinical utility of PD-L1 as a biomarker of response to anti-PD-1-based therapies has been limited by its heterogenous expression in melanoma,^16^ and the complex immunobiology underlying response and resistance. Therefore, while these suggested biomarkers have been shown to be associated with response, they lack the sensitivity and specificity to be utilized to guide treatment selection. Results from large, randomized Phase 3 trials including patients with unresectable Stage III or Stage IV metastatic melanoma revealed that patients treated with anti-PD-1 ± anti-CTLA-4 had better response rates, PFS and OS, compared to those treated with ipilimumab alone, regardless of PD-L1 expression, indicating that tumor PD-L1 positivity alone was not predictive of clinical outcomes.^17,18^

There is limited existing literature elucidating the role of novel drug target expression in clinical outcomes following treatment with novel immunotherapy combinations. In the randomized Phase 2 OpACIN-neo trial evaluating the efficacy of neoadjuvant ipilimumab + nivolumab in macroscopic stage III melanoma, high tumor mutational burden and high interferon-gamma-related gene expression were associated with pathologic response and low risk of relapse.^19^ However, tumor PD-L1 expression was not associated with pathological response to neoadjuvant immunotherapy.^20^

In the current study, we demonstrated a significant association between LAG-3 expression and response to combination anti-LAG-3 + anti-PD-1 immunotherapy. We observed LAG-3 positivity (LAG-3 expression ≥ 1%) in 60% of patients and found that these patients had a significantly longer PFS compared to those with LAG-3-negative tumors. This is in line with the findings from the RELATIVITY-047 trial which demonstrated LAG-3 positivity in 75% of patients, with longer PFS in nivolumab + relatlimab treated patients with LAG-3 expression ≥ 1% compared to those with < 1%.^3^ However, a similar trend was also observed in patients treated with nivolumab alone.^3^ Furthermore, a benefit was observed following treatment with nivolumab + relatlimab compared to nivolumab alone, regardless of LAG-3 or PD-L1 status (≥1% or < 1%).^3,4^ Interestingly, analysis of concurrent LAG-3 and PD-L1 expression revealed that patients with LAG-3+ PD-L1- tumors had the greatest benefit with combination nivolumab + relatlimab compared to nivolumab alone^4^. As all of the patients in our study were treated with combination anti-PD-1 and anti-LAG-3 therapy, we were not able to assess the impact of LAG-3 positivity on survival in patients treated with anti-PD-1 alone. Furthermore, in contrast to our findings, LAG-3 expression assessed via CyTOF analysis was not associated with response to neoadjuvant nivolumab and relatlimab combination immunotherapy in patients with resectable melanoma.^21^ Therefore, further studies are required to elucidate the predictive value of LAG-3 alone, and together with PD-L1, in early and advanced stage melanoma.

We also observed a significant association between LAG-3 expression and response and PFS in a subset of patients who had previously progressed following anti-PD-1 therapy. Similarly, in a cohort of patients with metastatic melanoma who received combination anti-LAG-3 and anti-PD-1 therapy following progression on anti-PD-1 therapy, the ORR was 14.1% in patients with LAG-3 expression ≥ 1% compared to 5.4% in patients with < 1%.^2^ Furthermore, in patients with previously treated unresectable/metastatic melanoma, two of three responders to the combination of spartalizumab + ieramilimab (anti-LAG-3) had LAG-3-positive tumors based on a positivity threshold of 5% staining.^22^ These findings suggest that immunohistochemical assessment of LAG-3 expression could potentially aid in the selection of second-line treatments for patients who progress on standard-of-care immunotherapies.

Immunohistochemistry scoring of LAG-3 at low percentage values (<5%) is often challenging and likely to be prone to intra and interobserver variability. In our study, 81% of patients showed < 5% LAG3 expression, with 40% of patients classified as negative (<1% positive staining). Interobserver variability is also likely to be present when attempting to assess the percentage of expression on immune cells only, to the exclusion of dendritic cells, and when attempting to qualitatively analyze the pattern of expression (e.g., membranous versus cytoplasmic) in such small cells. Artificial intelligence-related image assessments may improve this subjectivity in the future.

Our study assessed the expression of LAG-3 on immune cells in association with clinical outcomes, as per the LAG-3 scoring criteria in the RELATIVITY-047 trial^11^. While the current study used a different LAG-3 clone compared to the RELATIVITY-047 trial, both assays showed similar staining patterns, with punctate, cytoplasmic, and membranous LAG-3 expression observed on positive immune cells. LAG-3 assessment on TILs has also been commonly used to evaluate LAG-3 expression in various cancers including esophageal squamous cell carcinoma,^23^ colon cancer,^24^ and non-small cell lung cancer.^25^ Interestingly, overall LAG-3 expression correlated strongly with the presence of TILs, in keeping with previous findings.^26^ However, multivariate analyses revealed that LAG-3 expression and M stage were significantly associated with response to combination anti-LAG-3 + anti-PD-1 immunotherapy, independent of TILs. In addition to its expression on TILs, LAG-3 has also been shown to be expressed on dendritic cells in both mice^27,28^ and on plasmacytoid dendritic cells (pDCs) in humans.^29^ The unique characteristic and primary function of pDCs is the secretion of high levels of type 1 interferon.^30^ We noted the presence of LAG-3-positive cells showing dendritic cytomorphology; however, a more precise characterization of the cell lineage was not possible and represents a limitation of the study. LAG-3 was expressed at higher levels on plasmacytoid dendritic cells (pDCs) compared to effector T-cells or regulatory T-cells.^28^ Furthermore, the interaction between LAG-3 on human pDCs and MHC Class II on melanoma cells resulted in the activation of LAG-3+ pDCs and secretion of the cytokine IL-6, suggesting a role for LAG-3+ pDCs in driving immunosuppression in the melanoma microenvironment.^29^ These data highlight the need for further research into LAG-3 expression on dendritic cells and the potential role of this population in response or resistance to anti-LAG-3 immunotherapies.

## Conclusion

In conclusion, our study investigating LAG-3 expression in baseline melanoma specimens reveals the strong association between higher LAG-3 expression and response to combination anti-LAG-3 and anti-PD-1 immunotherapy. We further demonstrate an association between LAG-3 positivity and progression-free survival. Our findings add to current ongoing investigations to identify biomarkers of response to the recently approved anti-LAG-3 combination immunotherapy in metastatic melanoma.

## Supplementary Material

Supplemental Material

## Data Availability

The data that support the findings of this study are available from the corresponding author, G.V.L., upon reasonable request.
