# Supplementary figures and images for: Lag-3 expression and clinical outcomes in metastatic melanoma patients treated with combination anti-lag-3 + anti-PD-1-based immunotherapies
Source: Oncoimmunology. 2023 Oct 4;12(1):2261248. doi: 10.1080/2162402X.2023.2261248 (PMC10558007; doi:10.1080/2162402X.2023.2261248)

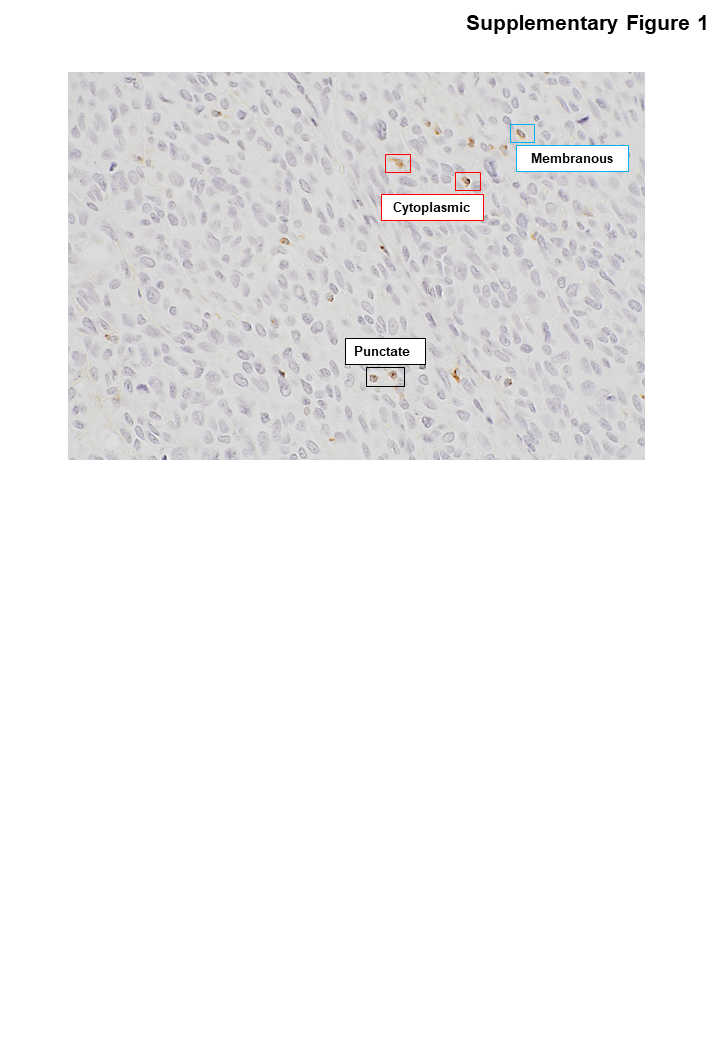

Supplement: Supplemental Material [file KONI_A_2261248_SM1298.tif]
